# Diagnostic and therapeutic targeting of pathological tau proteins in neurodegenerative disorders

**DOI:** 10.1002/2211-5463.13711

**Published:** 2023-09-30

**Authors:** Naruhiko Sahara, Makoto Higuchi

**Affiliations:** ^1^ Department of Functional Brain Imaging, Institute for Quantum Medical Sciences National Institutes for Quantum Science and Technology Chiba Japan

**Keywords:** disease‐modifying therapy, neuroimaging, tau filaments, tau PET, tauopathy

## Abstract

Tauopathies, characterized by fibrillar tau accumulation in neurons and glial cells, constitute a major neuropathological category of neurodegenerative diseases. Neurofibrillary tau lesions are strongly associated with cognitive deficits in these diseases, but the causal mechanisms underlying tau‐induced neuronal dysfunction remain unresolved. Recent advances in cryo‐electron microscopy examination have revealed various core structures of tau filaments from different tauopathy patients, which can be used to classify tauopathies. *In vivo* visualization of tau pathology is now available using several tau positron emission tomography tracers. Among these radioprobes, PM‐PBB3 allows high‐contrast imaging of tau deposits in the brains of patients with diverse disorders and tauopathy mouse models. Selective degradation of pathological tau species by the ubiquitin‐proteasome system or autophagy machinery is a potential therapeutic strategy. Alternatively, the non‐cell‐autonomous clearance of pathological tau species through neuron–glia networks could be reinforced as a disease‐modifying treatment. In addition, the development of neuroinflammatory biomarkers is required for understanding the contribution of immunocompetent cells in the brain to preventing neurodegeneration. This review provides an overview of the current research and development of diagnostic and therapeutic agents targeting divergent tau pathologies.

## Graphical abstract

For the establishment of biomarkers assisting tauopathy‐targeting therapies, we propose a strategy of neuron–glia interaction‐based drug development. Microglia pathological stages can be linked to tau pathology stages defined by tau positron emission tomography (PET) imaging for further selection of tauopathy‐related disease‐associated microglia markers. Currently, new neuroinflammatory PET ligands are urgently needed.

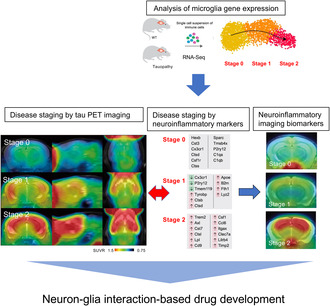

**Abbreviations**
AD: Alzheimer's disease
AGD: argyrophilic grain disease
AUTACs: autophagy‐targeting chimeras
BAG3: BCL2‐associated athanogene 3
CBD: corticobasal degeneration
CNS: central nervous system
cryo‐EM: cryo‐electron microscopy
CSF: cerebrospinal fluid
CTE: chronic traumatic encephalopathy
DAM: disease‐associated microglia
NFTs: neurofibrillary tangles
PET: positron emission tomography
PHFs: paired helical filaments
PiD: Pick disease
PROTACs: proteolysis‐targeting chimeras
PSP: progressive supranuclear palsy
SFs: straight filaments
TSPO: mitochondrial 18‐kDa translocator protein
UPS: ubiquitin‐proteasome system

Accumulation of intracellular neurofibrillary tangles (NFTs) consisting of microtubule‐associated protein tau is a major hallmark of Alzheimer's disease (AD) and related neurogenerative diseases, collectively referred to as tauopathies [1, 2, 3]. In AD, tau depositions are noted in neurons as somatodendritic NFTs, neuropil threads, and dystrophic neurites encompassing senile plaques, and the loss of neurons in specific regions coincides with the progression of NFTs [4]. The distribution of NFTs throughout the brain is correlated with the severity of cognitive impairment [5]. Hence, NFTs and/or tau assembly at an earlier fibrillogenesis stage are considered to be toxic species. Although the identity of the exact neurotoxic tau species remains unclear, studies of experimental models suggest that NFTs themselves may not be neurotoxic [6, 7, 8, 9]. NFT formation is initiated by the conversion of natively unfolded tau protein into insoluble tau aggregates through dimerization, oligomerization, and protofibril formation and is tightly associated with neurodegenerative processes. It is possible that a specific structural change of tau molecules from a physiological to a disease state provokes neurotoxicity at an early stage of neurodegeneration. The identification of cryo‐electron microscopy (cryo‐EM) structures of tau filaments from tauopathy brains may help to prove this hypothesis [10].

Nevertheless, it is essential to identify such a specific structure for therapeutic mitigation of neurotoxic insults. On the other hand, similar to other cerebral proteinopathies that are characterized by the existence of aggregated forms of proteins or peptides (e.g., amyloid‐β, tau, α‐synuclein, and TDP‐43), dissecting aggregation processes may provide a universal strategy for the development of disease‐modifying treatments against AD. Unlike other amyloid‐forming proteins, tau protein does not undergo fibrillization *in vitro* without any inducer molecules (e.g., heparin, heparan sulfate, polyunsaturated fatty acid, RNA, or quinones). At present, the endogenous, physiological inducers of tau aggregation are not known yet and need to be discovered for unraveling the initiation of NFT formation.

Neurotoxic tau species may provoke neuronal cell death in a cell‐autonomous manner. In general, intracellular protein degradation is important for the maintenance of protein metabolism and for preventing the accumulation of misfolded proteins [11]. Tau protein metabolism is typically maintained by ubiquitin‐proteasome system (UPS) and autophagy‐lysosome systems [12, 13]. Reducing pathological tau accumulation through these protein degradation systems could be one of the therapeutic strategies, although it remains elusive whether these systems can selectively process pathological but not physiologically functioning tau species.

Microglia are the resident phagocytes of the central nervous system (CNS), and their activation is considered to play an important role in the pathogenesis of neurodegenerative diseases. Recent studies with single‐cell RNA sequencing analysis of CNS cells in AD and other neurodegenerative conditions revealed that the transition from homeostatic microglia to disease‐associated microglia (DAM) was defined by changes in the expression of characteristic genes [14, 15]. However, it is yet to be clarified whether and when changes in gene expression occur in response to the pathogenesis. Non‐cell‐autonomous mechanisms governed by DAM should be taken into account for preventing neurotoxicity injuries and death of pathological tau‐bearing neurons.

The current research conducted on cryo‐EM has revealed distinctive structural variations in the tau fibril core in diverse tauopathies. Furthermore, tau positron emission tomography (PET) imaging has demonstrated its potential in diagnosing tauopathies by analyzing the pathological distribution of tau deposits in living brains. In this review, we overview the recent research progress to clarify diverse tau assemblies and their implications for the diagnosis of various tauopathies. Additionally, we present insights into the degradation systems acting on pathological tau species from the perspective of protein homeostasis. Finally, we discuss the potential link between tau‐induced neurodegeneration and microglial functions, as microglia play a crucial role in maintaining brain homeostasis and promoting neuronal deteriorations and may be a key target for disease‐modifying therapies.

## The tau protein

**Fig. 1:**
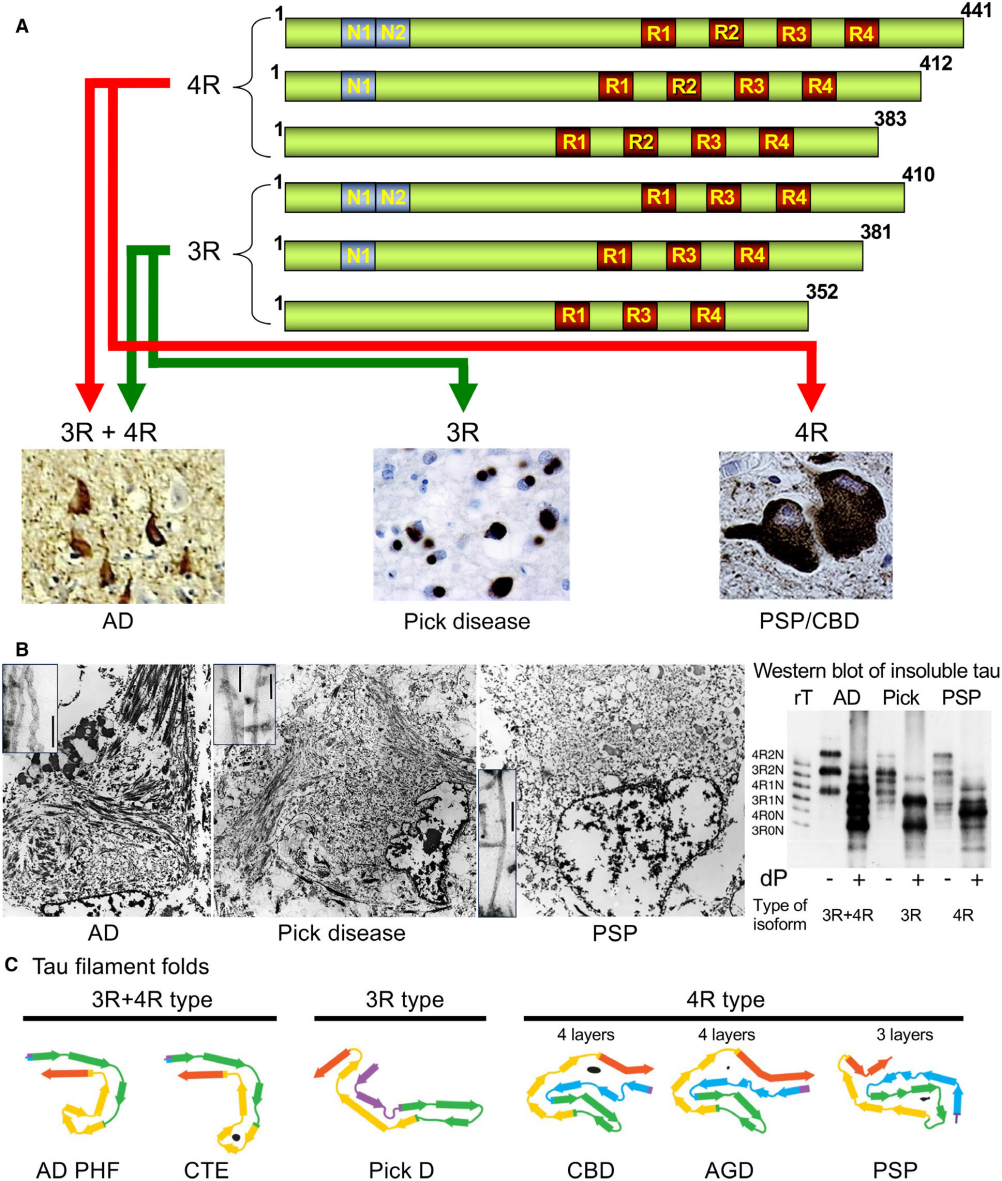
Disease‐associated characteristics of tauopathies. (A) Tauopathies can be classified into three groups: 3R + 4R tauopathies, including AD, 3R tauopathies, including PiD and 4R tauopathies, including PSP and CBD. (B) Representative electron microscopical images of tau assemblies in AD, PSP, and PiD brains. Inboxes show PHFs and SFs in AD, SFs and twisted filaments in PSP, and SFs with some wide twisted filaments in PiD. (C) The core structures of tau filaments observed by cryo‐EM examination. AD and CTE are classified into 3R + 4R type [10]. PiD is classified into 3R type. CBD, AGD, and PSP are classified into 4R type. The 4R types are divided into two classes. The PSP tau filament fold comprises three‐layered core regions, whereas filamentous structures from CBD and AGD brains are four‐layered folds.

The tau protein belongs to the family of Tau/MAP2/MAP4 microtubule‐associated proteins. In the CNS, tau is mostly expressed in neurons but is also present at low levels in glia [16]. Alternative splicing of MAPT in the adult human brain generates six tau isoforms [17] (Fig. 1A). These isoforms range from 352 to 441 amino acids in length and differ by the presence or absence of inserts of 29 or 58 amino acids (encoded by exons 2 and 3) in the N‐terminal half, and the inclusion or exclusion of the 31 amino acid microtubule‐binding repeat (encoded by exon 10) in the C‐terminal half [18] (Fig. 1A). The inclusion of exon 10 results in the production of three tau isoforms with four microtubule‐binding repeats (4R) and its exclusion in other three isoforms with three repeats (3R). In the adult human brain, similar levels of 3R and 4R tau isoforms are expressed [19]. Tauopathies can be classified into the following three groups on the basis of the tau isoforms found in the aggregates from diseased brains: (1) 4R tauopathies, including progressive supranuclear palsy (PSP), corticobasal degeneration (CBD) and argyrophilic grain disease (AGD); (2) 3R tauopathies, including Pick disease (PiD); and (3) 3R + 4R tauopathies, including AD and chronic traumatic encephalopathy (CTE) [20, 21] (Fig. 1A–C). Dominantly inherited mutations in MAPT cause a form of frontotemporal dementia that can be associated with parkinsonism (FTDP‐17T). To date, more than 80 mutations have been identified in either exonic or intronic regions of human MAPT (Alzforum website) and can be classified into missense and splicing mutations. The majority of missense mutations cluster around the microtubule‐binding domain. Most splicing mutations are within or near intron 10, increasing the inclusion of exon 10 and consequently the ratio of 4R tau to 3R tau, although there are several exceptions such as ΔK280, L266V, and G272V mutations that increase 3R versus 4R tau isoforms. In human brains, the imbalance between the 3R and 4R tau may be a key event to cause tauopathies, but the mechanisms underlying this alteration are still unknown. Importantly, tau isoform expression is not conserved between species [22, 23]. Adult mice express predominantly 0N4R isoform, while rats have 0N4R, 1N4R, and 2N4R with the same ratios [24]. In mice, 3R isoform is mainly expressed during the embryonic stage and is replaced by 4R tau between postnatal days 9 and 18 [25]. This isoform switching most likely induces axonal elongation and neuronal development.

To search physiological functions of tau, several lines of tau‐knockout mice were extensively examined [26]. Early investigations showed that tau‐knockout mice presented no overt abnormal phenotypes [27, 28, 29]. However, recent studies have revealed several pathological changes with behavioral abnormality in tau‐knockout mice [30, 31, 32]. In contrast to the indispensability of tau, deficiency of tau protected against Aβ‐induced excitotoxicity [33, 34], indicating the involvement of endogenous tau in regulating neuronal activity under pathological circumstances. However, it is still unknown whether tau plays a significant role in physiological network activity because these experiments were performed under excessive expressions of the human amyloid precursor protein. In addition, research on tau‐knockout mice has shown that tau may be involved in neurogenesis [35], iron export [31], and long‐term depression (LTD) [36].

## Neuropathological features of tau inclusions

Although tauopathies share a common molecular mechanism, histopathological features vary across disease types [37]. Pathological features of AD brains are NFTs composed of paired helical filaments (PHFs) and straight filaments (SFs). Immunostaining of AD brain slices with antibodies against phosphorylated tau (p‐tau) labels NFTs, neuropil threads, and dystrophic neurites surrounding Aβ deposits (i. e. neuritic plaques). PSP, CBD, and PiD are non‐AD tauopathies with focal cortical and/or subcortical neuronal loss and gliosis. These non‐AD tauopathies are categorized in the spectrum of sporadic frontotemporal lobar degeneration with tau pathology (FTLD‐tau). Pathological features of PSP brains are globose NFTs, tufted astrocytes, and oligodendrocytic coiled bodies observed in the subthalamic nucleus, globus pallidus, ventral thalamus, cerebellar dentate nucleus, and cerebral cortex [38]. Ultrastructural assessments showed that tau assemblies in PSP were mostly composed of SFs with rare twisted filaments (Fig. 1B). CBD is one of the 4R tauopathies with neuronal and glial hyperphosphorylated tau aggregates in both gray and white matter of the neocortex, basal ganglia, thalamus, and the brainstem [39]. Astrocytic plaque is a unique glial pathology in CBD. Ultrastructures of tau aggregates in CBD are mostly SFs with some wide twisted filaments. Either PSP and CBD can be found as a neuropathological background in diverse FTLD syndromes, including corticobasal syndrome, PSP syndrome, frontotemporal dementia, and nonfluent/agrammatic primary progressive aphasia [40, 41, 42]. Pick bodies forming round intraneuronal inclusions are the histopathological hallmark of PiD [43, 44]. These inclusions are composed of hyperphosphorylated 3R tau and are typically observed in hippocampal pyramidal neurons and granular neurons of the dentate gyrus. There are ballooned neurons and variable tau‐immunoreactive glial inclusions [45]. Ultrastructures of tau aggregates in PiD are mostly SFs with some wide twisted filaments (Fig. 1B). In AGD brains, grains are typically found in the neuropil of limbic areas and diffusely deposited in the cortex. Spindle‐shaped 4R tau lesions in neuronal processes, coiled bodies in oligodendrocytes, and pretangles in neurons are also present in AGD brains [37, 46]. Neuropathological features of CTE are defined by the irregular cortical distribution of p‐tau immunoreactive NFTs and astrocytic tangles with a prediction for the depth of cerebral sulci. 3R + 4R tauopathy was confirmed by immunohistochemistry [47] and biochemistry [48]. Ultrastructures of CTE tau filaments are predominantly helical filament type with projected widths of 20–25 nm and crossover spacings of 65–80 nm [48]. These filaments differ from the PHFs and SFs of AD.

Current advances of cryo‐EM examination revealed that tauopathies could be classified according to the core structures of tau filaments [10]. Even if components of tau aggregates are similar between AD and CTE, the CTE tau filament fold is distinct from that of the AD tau filament (Fig. 1C). Based on cryo‐EM observations, the 4R tauopathies are divided into two classes [10]. The PSP tau filament fold comprises three‐layered core regions, whereas filamentous structures from CBD and AGD brains are four‐layered folds (Fig. 1C). The differences in the filament structures between these 4R tauopathies are consistent with the profiles of N‐terminally truncated tau fragments examined by western blots of sarkosyl‐insoluble tau [10].

Because tauopathies induce a broad range of symptoms such as behavioral, movement, language, and memory impairments [49, 50], the clinical diagnosis and differentiations of these illnesses may not necessarily be in agreement with neuropathological classifications. The definitive diagnosis of tauopathies has been only enabled by examining the shapes and distribution of tau deposits, affected cell types, and tau isoform composition in autopsied brain samples. Now, cryo‐EM assays can identify disease‐characteristic filamentous structures of tau inclusions from postmortem brains, despite the lack of histological information due to the extractions of buffer‐insoluble materials from tissues with a large volume. Such ultrastructural properties can also be associated with subcellular, cellular, and regional localizations and morphology of inclusions.

## Neuroimaging‐based diagnostic assessments of tauopathies

It is known that high levels of p‐tau181 (tau phosphorylated at Thr181) and total tau have consistently been found in cerebrospinal fluid (CSF) of AD patients relative to healthy elderly controls [51]. Since CSF p‐tau181 levels in AD are higher than those in non‐AD tauopathies, CSF p‐tau181 is also a better indicator for differential diagnosis [52]. Recent reports showed that relative levels of p‐tau217 (tau phosphorylated at Thr217) in CSF were correlated with burdens of PET‐detectable Aβ and tau aggregates and CSF measures of Aβ [53, 54]. Janelidze et al. also observed that CSF p‐tau217 correlates stronger than CSF p‐tau181 with PET measures of tau and amyloid pathologies in AD and hypothesized that p‐tau217 levels may reflect the pathological state of tau better than p‐tau181 levels, although the sensitivity of p‐tau is highly dependent on the performance of antibodies (e.g., anti‐p‐tau217 antibody IBA413 and anti‐p‐tau181 antibody AT270) [54]. More recently, it was reported that plasma p‐tau (both p‐tau181 and p‐tau217) was able to discriminate AD from healthy control and FTLD [55, 56, 57]. Compared with CSF analyses, blood‐based biomarkers can be widely used in primary clinical settings as less invasive and equally cost‐effective tools.

Imaging biomarkers are now available for detecting *in vivo* tau pathology with a panel of PET tracers. Tau tracers are designed according to β‐sheet binding properties and labeled AD‐type tau deposits, while these compounds exhibit differential reactivities with non‐AD tau aggregates. By now, [^11^C]PBB3, [^18^F]PM‐PBB3, [^18^F]AV1451, [^18^F]THK5351 (and its analogs), [^18^F]MK‐6240, [^18^F]R06958948, [^18^F]GTP‐1, and [^18^F]PI‐2620 have been applied to human subjects [58, 59, 60, 61, 62, 63, 64, 65] (Table 1). The distribution of the bound tracers recapitulated Braak NFT staging in AD [66]. First‐generation tracers exemplified by [^18^F]AV1451 and [^18^F]THK5351 exhibit off‐target effects such as binding to monoamine oxidase (MAO)‐A and MAO‐B [67, 68], whereas second‐generation tracers such as [^18^F]PM‐PBB3, [^18^F]MK‐6240, [^18^F]R06958948, [^18^F]GTP‐1, and [^18^F]PI‐2620 seem to have less off‐target binding [61, 69, 70]. [^11^C]PBB3 was designed to capture tau deposits in a wide range of tauopathies [58]. This ligand reacts with 3R and 4R tau pathologies in human brains better than [^18^F]AV1451 [71]. [^18^F]PM‐PBB3, which was generated by modifying the chemical structure of PBB3 for relatively high metabolic stability, has the advantage of an ^18^F‐labeled probe over ^11^C‐radiotracers for broader availability and higher PET scan throughput. At present, [^18^F]PM‐PBB3 is the unrivaled tracer to capture diverse tau fibrils with different isoform compositions and conformations with contrast and dynamic range adequate for individual‐based assessments of AD‐ and FTLD‐spectrum syndromes [65]. Although the feasibility of fluid biomarker tau PET imaging for evaluating the severity of tau pathologies in AD patients still needs to be established by examining their correlation with neuropathological findings in postmortem assessments, the relationship between these two bioassay modalities has been indicated. Indeed, a mass‐spectrometry‐based assay recently demonstrated that the microtubule‐binding region of tau containing the residue 243 (MTBR‐tau243), p‐tau205 and p‐tau217 in CSF were associated with [^18^F]AV1451‐PET imaging and cognitive deficits [72].

**Table 1:** Properties of tau PET tracers. Relative performance of each radioprobe was indicated according to contrasts for AD tau lesions. Some of radioprobes detected *in vivo* tau pathology of non‐AD tauopathies. Monoamine oxidase (MAO)‐A, MAO‐B and choroid plexus (CP) are known as off‐targets of several tau PET tracers.

PET tracer	Developer	Contrast for AD tau lesions	Non‐AD tauopathy	Off‐target binding	
				MAOs	CP
[^11^C]PBB3	QST	1.2–1.4	4‐repeat tauopathy 3‐repeat tauopathy	−	±
[^18^F]AV1451	Avid/Lilly	1.3–1.6	4‐repeat tauopathy (low contrast)	MAO‐A	+
[^18^F]THK5351	Tohoku Univ.	1.4–1.8	4‐repeat tauopathy	MAO‐B	−
[^18^F]PI‐2620	Piramal	2.0–2.5	4‐repeat tauopathy	−	−
[^18^F]GTP1	Genentech	2.0–3.0		−	−
[^18^F]RO6958948	Roche	2.0–2.5		−	−
[^18^F]MK‐6240	Merk	2.0–3.0		−	−
[^18^F]PM‐PBB3	QST APRINOIA	2.0–2.5	4‐repeat tauopathy 3‐repeat tauopathy	−	+

**Fig. 2:**
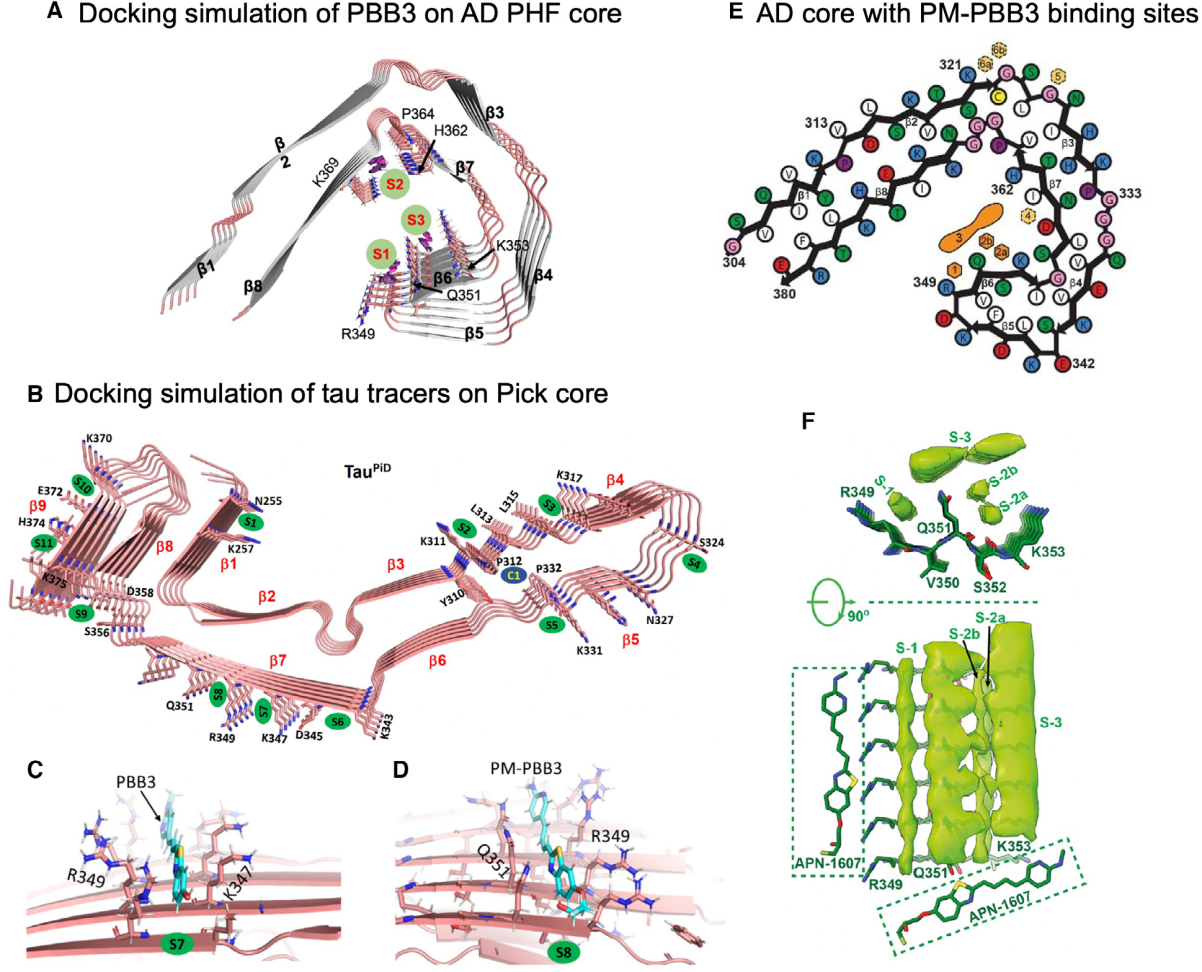
Binding sites of tau PET probes in tau filament core. (A) Molecular docking of PBB3 into the PHF protofilament core structure [74]. S1 site has the highest affinity for the tau filaments, followed by S2 and S3. (B) Binding sites of tau PET tracers in Pick filament core predicted by molecular docking (MD) [115]. Secondary structures of Pick filaments (K254 to F378) show binding sites S1 to S11 (green) and cavity site C1 (blue). (C, D) Molecular interactions of PBB3 (C) and PM‐PBB3 (D) with Pick filaments taken after 100‐ns MD simulation [115]. PBB3 binds to R349 and K347 sidechain hydrogens. PM‐PBB3 binds to R349 and Q351 sidechain hydrogens. (E) AD core with PM‐PBB3 binding sites [76]. Major binding sites 1, 2a, 2b and 3 are shown in orange. Minor binding sites 4, 5, 6a and 6b are indicated in yellow. (F) Binding of PM‐PBB3 to SFs [76]. Top views and side views of extra densities in the PM‐PBB3 binding sites.

The binding properties of tau PET tracers with tau fibrils are examined by computational modeling using structural information from cryo‐EM studies [73, 74] (Fig. 2A–C). The modeling identified several potential high‐affinity binding sites with some diversities of binding reactivity for each tracer [73, 74]. To clarify whether different tau filamentous structures can be distinguished by tau PET tracers, Mishra et al. performed molecular modeling study on tau PET tracer binding to the core structure of PiD tau filament [75] (Fig. 2B–D). The examined tracers, including AV‐1451, MK‐6240, PBB3, PM‐PBB3, THK5351, and PiB, bind to PiD tau filament fold at multiple surface binding sites and in a cavity binding site. Docking and molecular dynamics simulations revealed a unique binding site (the groove between R349 and Q351) for PBB3 and PM‐PBB3 (Fig. 2C,D). To further confirm PM‐PBB3 binding on the tau filament fold, a cryo‐EM examination of the AD tau filament fold with PM‐PBB3 was performed [76] (Fig. 2E,F). One of the two major binding sites was a groove between R349 and Q351, which commonly existed in PHFs and SFs. The groove between the side chains of Q351 and K353 contains two binding sites, and there are side‐to‐side interactions between PM‐PBB3 compounds in parallel with the long helical axis of tau filaments (Fig. 2F). Interestingly, an additional binding site in a direction nearly perpendicular to the helical axis in SFs was observed though resolutions did not reach to define the orientation of PM‐PBB3 (Fig. 2F). Due to less space in the cavity, perpendicular bindings of PM‐PBB3 were not present in PHFs. Assumedly, perpendicular bindings of PM‐PBB3 forming a high‐density ladder amplify PET signals. Thus, tau fibrils in non‐AD tauopathies such as PSP, CBD, CTE, and PiD could have similar properties of PM‐PBB3 binding. It will be essential to identify the binding sites of PM‐PBB3 in tau filaments from these non‐AD tauopathies by cryo‐EM examination in the future. Nevertheless, cryo‐EM is a promising research tool for developing new PET tracers with higher specificity and affinity towards the high‐precision differential diagnosis of tauopathies.

## Targeting tau protein homeostasis for therapies

**Fig. 3:**
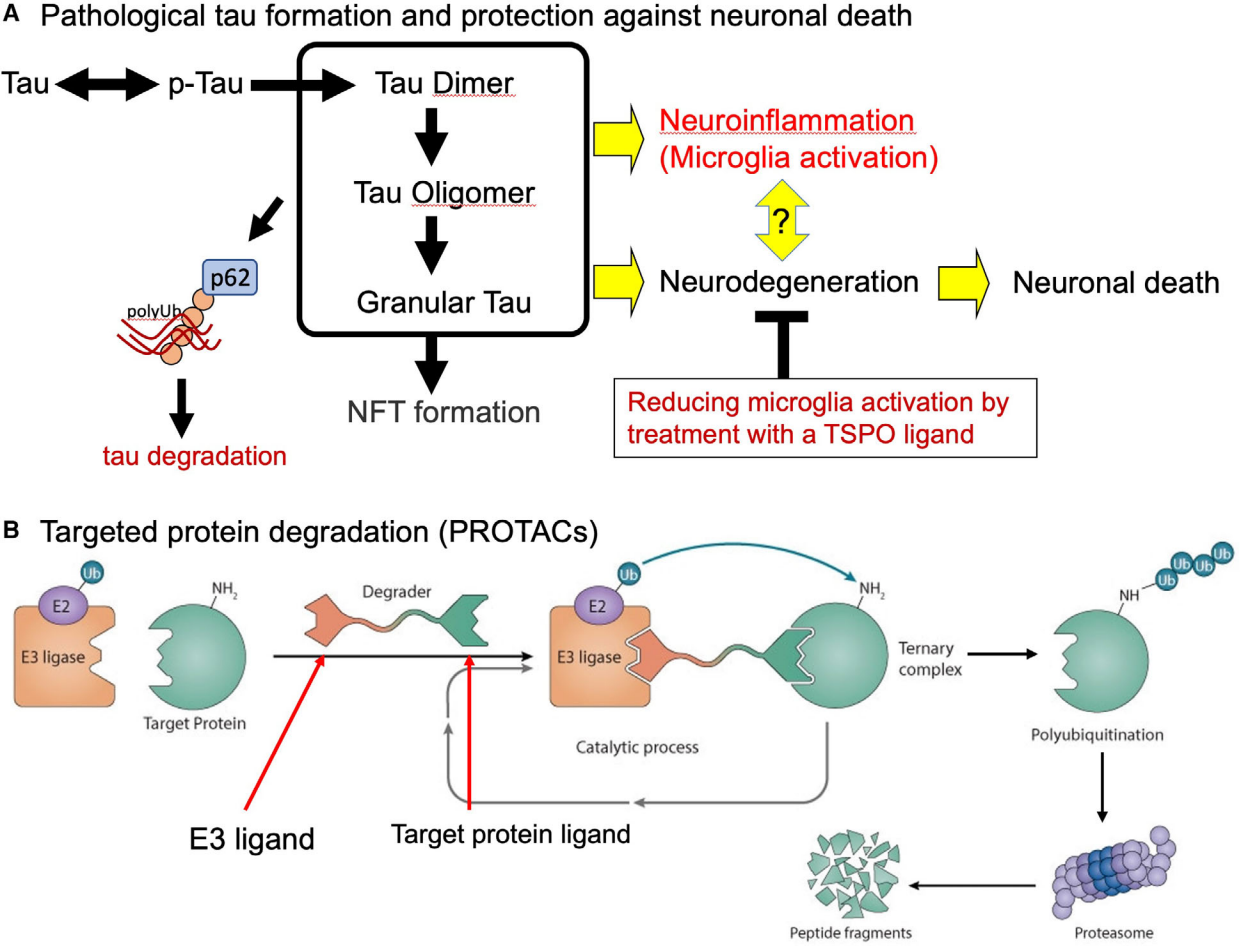
Therapeutic targets for preventing tauopathies. (A) Process of pathological tau formation and neuronal death. The autophagy receptor p62 may trigger tau degradation by eliminating neurotoxic tau species. TSPO ligand Ro5‐4864 may attenuate both microglia activation and neurodegeneration. (B) The PROTACs is one of the novel technologies for eliminating neurotoxic components. A degrader binds to target misfolded proteins and E3 ubiquitin ligase to induce Lys48 polyubiquitination and proteasomal degradation of the target protein.

In general, the endoplasmic reticulum system, autophagy‐lysosome system, and UPS are the three main regulatory pathways for maintaining protein homeostasis and preventing excess dysfunctional protein species. Several research groups have focused on the identification of pathways involved in the degradation of misfolded tau proteins, since such molecular machineries are likely to be impaired in the pathogenesis of tauopathies and are of critical significance as targets for disease‐modifying therapies. Duff and her colleagues reported the selective vulnerability of excitatory neurons to tau pathology and observed higher levels of BCL2‐associated athanogene 3 (BAG3), a facilitator of autophagy, in inhibitory neurons than in excitatory neurons [77]. Insufficient clearance of excessive tau protein may cause excitatory neuronal cell death. These excessive tau species may form toxic conformers. As mentioned above, NFTs may not be the primary toxic tau species in the brains of patients with AD and other tauopathies [6], and researchers hypothesized that tau oligomers are responsible for a large part of disease‐related neurotoxicity [78, 79]. When several tau species were injected into the mouse hippocampus, tau oligomers caused memory deficits and cell damage, while neither tau monomers nor tau fibrils caused any abnormality [80]. Recently, we reported that the genetic ablation of p62/SQSTM1, a ubiquitinated cargo receptor for selective autophagy, exacerbates tau pathologies, neuronal death, and neuroinflammation in a mouse model of tauopathy (PS19 mice) [81]. Immunolabeling analyses with an antibody selectively recognizing tau oligomers [82] demonstrated that PS19/p62‐KO mice displayed accumulation of tau oligomers at a significantly high level than PS19 mice. Since p62 is the most abundant and major autophagy receptor in mouse brains [81], p62 likely exerts neuroprotection against tau pathologies by eliminating neurotoxic tau species (Fig. 3A).

The proteolysis‐targeting chimeras (PROTACs), which bind to target misfolded proteins and E3 ubiquitin ligase to induce Lys48 polyubiquitination and proteasomal degradation of the target protein, is one of the novel technologies for eliminating neurotoxic components [83] (Fig. 3B). Chu et al. showed that tau‐degrading PROTAC generated by fusing a tau‐binding motif from β‐tubulin to E3 ligase‐binding peptides promote tau degradation in an AD mouse model, 3xTg‐AD [84]. Another study documented a small‐molecule PROTAC composed of tau PET tracer, AV‐1451, and thalidomide, a ligand for Cereblon (CRBN, a substrate‐receptor for the E3‐ubiquitin ligase CRL4^CREN^) [85]. The concept of this study was to target disease‐associated tau and to reduce the stress vulnerability of FTD neurons. In addition to PROTAC, autophagy‐targeting chimeras (AUTACs) to degrade target proteins through autophagy machinery have been developed. Takahashi et al. reported that AUTACs contained guanine‐derived *p*‐fluorobenzylguanine tag and Halo Tag (HT) ligand selectively degraded target proteins through Lys63‐linked ubiquitination [86]. Taken together, PROTAC or AUTAC is a heterobifunctional peptide or small molecule that simultaneously binds to target proteins and UPS components or autophagy machinery to remove those proteins or dysfunctional organelles such as mitochondria [83, 86]. Targeting ubiquitin signaling could be a fundamental strategy for ameliorating neuronal function and survival.

## Targeting neuron–glia interactions for therapies

Microglia are the resident phagocytic cells in the CNS and play a critical role in pathological and physiological processes. Microglia express a wide range of receptors that act as molecular sensors recognizing intracellular and extracellular insults followed by immune responses. Activated microglia become highly motile, secreting inflammatory cytokines and phagocytosing cell debris or damaged neurons [87]. To investigate the link between microglial activation and tauopathy, the tauopathy mouse model PS19 crossbred with TREM2 knockout mice was examined [88]. The lack of TREM2 rescued brain atrophy and decreased expressions of DAM markers such as APOE and Cst7, implying the critical involvement of DAM in tau‐induced neurodegeneration. It is also noteworthy that PET imaging of tauopathy mouse models, PS19 and rTg4510, showed an age‐dependent increase of the mitochondrial 18‐kDa translocator protein (TSPO), a marker of microglial activation, along with pathological tau accumulation and brain atrophy [89, 90]. These data raise a possibility that the upregulation of TSPO is associated with the deleterious roles of DAMs in these models. To investigate the potential of TSPO as a therapeutic target, the effects of treatment with a TSPO ligand, Ro5‐4864, were examined on the rTg4510 mice [91]. Ro5‐4864 treatment attenuated brain atrophy, hippocampal neuronal loss, and levels of C1q, a regulator of the complement cascade [91] (Fig. 3A). In another study, the elimination of a complement, C3, in PS19 mice was performed to investigate possible linkage between complement pathway and tau‐induced neurodegeneration [92]. C3 knockout in the PS19 mice ameliorated neuron loss and brain atrophy and improved neurophysiological and behavioral measures [92]. As Hansen et al. reported in their review article, reducing complement activation could be a potential therapeutic approach to tauopathies [93].

It is still unclear whether microglial phagocytosis plays a beneficial or detrimental role in neurodegenerative diseases. However, effective clearance of neurotoxic components could be a potential therapeutic strategy for preventing neurodegenerative diseases. There are a number of receptor systems contributing to phagocytosis in the CNS [94]. The TAM receptor tyrosine kinases, consisting of Tyro3, Axl, and Mer, have a critical function in macrophages and immune sentinels for the phagocytosis of apoptotic cells [95, 96, 97]. TAM receptors recognize phosphatidylserine exposed on the cell surface, an eat‐me signal, via protein S or GAS6 [98]. A recent study revealed that TAM receptors, Axl and Mer, were required for microglial recognition and phagocytosis of Aβ plaques [99]. Microglial gene expression analysis in neurodegenerative disease mouse models, including *App* ^*NL‐G‐F/NL‐G‐F*^, rTg4510, and SOD^G93A^ mice, showed upregulation of *Axl*, but no change of *Mer* in all three mouse models [100]. Hence, Axl could become a universal receptor for maintaining brain homeostasis by phagocytosis. Further characterization of TAM receptor‐associated eat‐me signaling in tauopathy will be necessary.

As a counterpart of DAM, the abundance of homeostatic microglia reflects the physiological, non‐diseased status. Transcriptome analysis of microglia isolated from neurodegenerative disease models showed a reduction of homeostatic genes *P2RY12*, *Tmem119*, and *CX3CR1*. [14, 100] In our hand, the rTg4510 mice showed the regression of P2RY12 protein level before the massive accumulation of intraneuronal tau deposits and an elevation of TSPO immunoreactivity [101]. Since P2RY12 declines preceded increases of Iba1 and TSPO in the rTg4510 mice, this homeostatic marker would be a sensitive marker heralding the activation of deleterious microglia. A recent study showed neuroprotective functions of microglia through somatic neuron–microglia interaction by P2RY12 clustering in the microglial process [102]. The cellular interaction was regulated by the purinergic signaling from neuronal mitochondria. Modulation of neuronal mitochondrial activity and/or purinergic signaling could lead to the maintenance of microglial homeostasis, although further studies should be performed to understand the significance of neuron–microglia interactions.

## Neuroimaging‐based drug development for preventing tauopathy

The drug development process should include target identification, drug screening, non‐clinical tests in animal models, and clinical trials [103]. The establishment of evaluation tools is of vital significance for adequate assessments of drug efficacies. In AD drug development, several biomarkers, such as brain imaging and CSF measures, can assist in diagnosis, demonstrate target engagement, and support disease modification. Of particular interest are imaging biomarkers, including amyloid PET and tau PET, as mentioned above. Tau PET tracers, [^11^C]PBB3 and [^18^F]PM‐PBB3, are able to capture wide‐range tau pathologies in AD, non‐AD tauopathies, and model mice exemplified by rTg4510 and PS19 [58, 65, 90, 104]. Importantly, *in vivo* brain imaging enables longitudinal examinations of neurodegenerative processes and pathological tau formation [105]. The use of animal models that recapitulate the critical features of the disease, such as NFTs, cognitive impairment, brain atrophy, and neuronal loss, greatly facilitates the evaluations of tau‐targeting therapies. Recently, a couple of neuroimaging‐based studies successfully demonstrated the efficacy of a TSPO ligand, Ro5‐4864, and a low‐protein diet on the rTg4510 mice [91, 106]. These studies showed suppression of brain atrophy measured by volumetric magnetic resonance imaging but no significant change of tau PET signals, suggesting that the neuroprotective effects of these treatments were on the downstream of tau accumulation. However, due to lower spatial resolution and partial volume effects in animal PET imaging, caution is warranted in interpreting findings in PET imaging of tauopathy mouse models. Moreover, radiosignals arising from off‐target tracer binding [107] need to be considered in the analysis of non‐clinical tau PET data.

There are limited numbers of animal models that display intracellular filamentous tau aggregations [108]. As far as we know, several lines of P301L/S mutant tau‐expressing transgenic mice developed neurofibrillary pathology in the CNS, whereas most non‐mutant tau‐expressing transgenic mice rarely developed tau pathology. Recent studies suggest that there is a non‐tau factor to induce neurodegeneration in the rTg4510 mice expressing P301L human tau due to transgene integrations into the coding sequence of mouse endogenous genes [109, 110]. Therefore, candidate drug interventions with current mouse models of tauopathy need to pay close attention to whether effects are really associated with tau‐induced neurodegeneration.

**Fig. 4:**
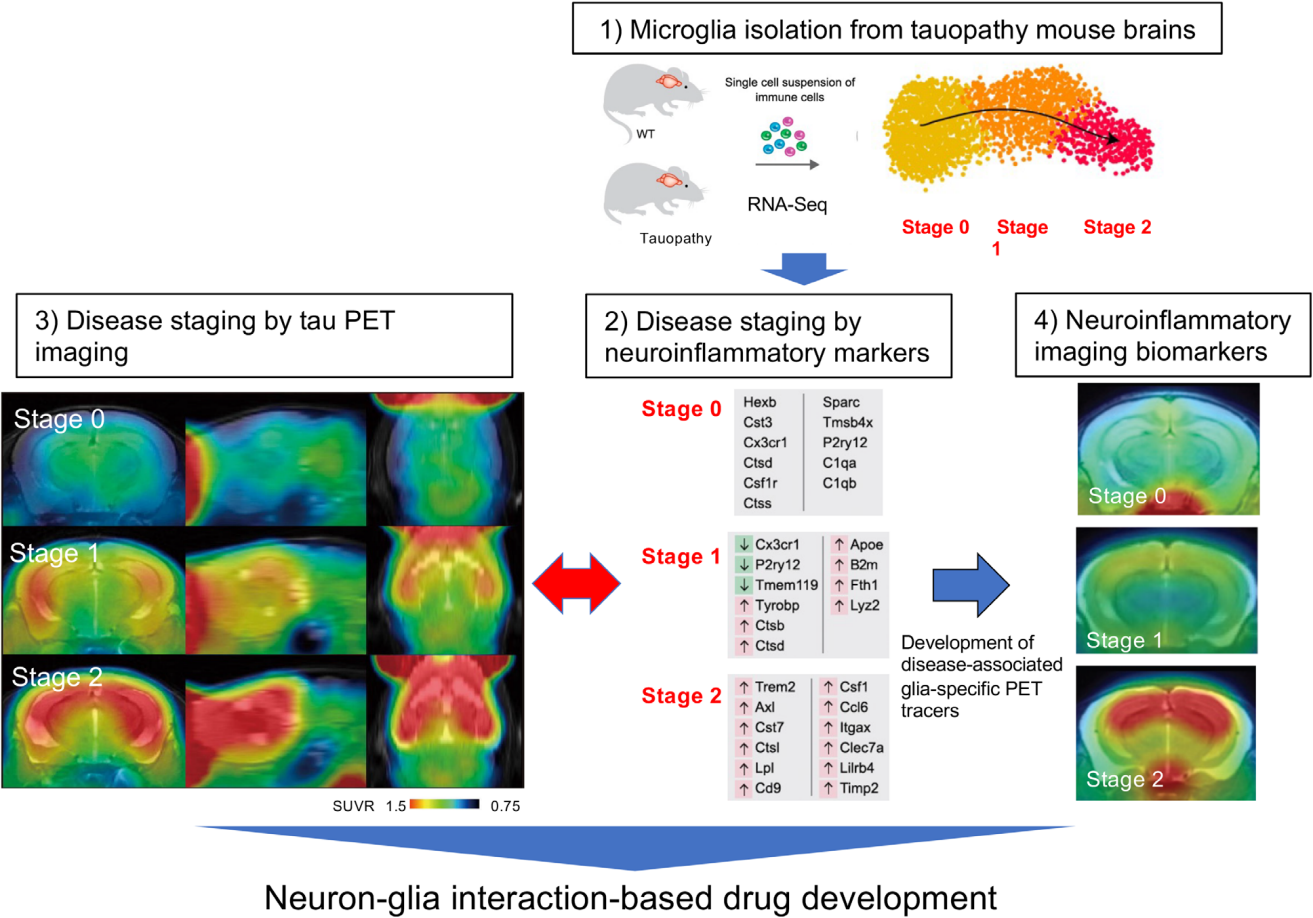
Schematic representation of neuron–glia interaction‐based drug development. For the establishment of biomarkers assisting tauopathy‐targeting therapies, we propose a workflow consisting of the following elements: (1) Microglia are isolated from WT and tauopathy model mice at different ages for single‐cell RNA‐seq gene expression profiling. (2) The DAM signature in tauopathy mouse models is examined for staging the tau‐related neurodegenerative phenotype of microglia, and candidates of neuroinflammatory biomarkers can be selected from stage‐dependent gene expressions. (3) Microglia pathological stages can be linked to tau pathology stages defined by tau PET imaging for further selection of tauopathy‐related DAM markers, and this approach can be supplemented by immunohistochemical and biochemical assays of excised tissues. (4) Selective small‐molecule, brain‐entering PET radioligands for the selected molecular targets are developed by *in‐vivo* imaging and *in‐vitro* autoradiographic evaluations. PET images of tau pathologies and neuroinflammatory changes in this figure tentatively illustrate the performance of ^18^F‐PM‐PBB3 and translocator protein (TSPO) tracer, ^11^C‐Ac5216, respectively, in rTg4510 mouse brains putatively at three different stages. However, new neuroinflammatory PET ligands will need to be generated as TSPO is not included in DAM makers.

Neuroinflammation is an inflammatory response in the CNS. The chronic over‐activation of pro‐inflammatory response has been implicated in many neurodegenerative diseases [111]. The development of neuroinflammatory biomarkers is necessary to understand the contribution of such responses to the initiation and progression of tauopathies. *In vivo* visualization of activated microglia in humans and mice is available with the use of PET tracers of TSPO [90, 112, 113]. *In vivo* imaging study of rTg4510 mice revealed that the increase of TSPO signal was a late event following pathological tau accumulation [90, 114]. To seek an early phenotypic change of microglia, other biomarkers will be needed. Currently, the DAM signature defined by RNA‐seq analysis in 5xFAD mice [14] was generally accepted for staging the neurodegenerative phenotype of microglia. Once the DAM signature in the rTg4510 mice is discriminated, microglia stages can be linked to pathological stages defined by tau PET imaging (Fig. 4). Candidates of neuroinflammatory biomarkers can be selected from microglia stage‐dependent gene expressions (Fig. 4). Neuroinflammatory biomarkers identified in mouse models can be translated to humans, otherwise reverse translational research can be conducted to re‐screen biomarkers. Eventually, these biomarkers will become powerful tools for drug development based on neuron–glia interactions.

## Conclusion

To achieve disease‐modifying therapies for tauopathies, it is crucial to identify an appropriate target to modulate the disease progression. The formation of local NFTs is strongly linked to cognitive impairment, and as tau protein is a main constitute of NFTs, it should be a primary target for the development of drugs and diagnostics. Recent advancements in cryo‐EM have enabled the identification of core structures of tau filaments in tauopathy brains, which can be used to classify different types of tauopathies. Tau PET imaging also holds the potential for diagnosing tauopathies by evaluating the distribution of pathological tau deposits in living brains. Additionally, interventions in protein degradation systems such as the UPS and autophagy‐lysosome system, as well as microglial phagocytosis, are currently being investigated for their effectiveness as disease‐modifying therapies.

## Conflict of interest

MH hold patents on compounds related to the present report (JP 5422782/EP 12884742.3/CA2894994/HK1208672).

## Author contributions

NS conceived and designed the project, NS and MH wrote the paper.

## References

[1] Lee VM , Goedert M and Trojanowski JQ (2001) Neurodegenerative tauopathies. Annu Rev Neurosci 24, 1121–1159. 11520930 10.1146/annurev.neuro.24.1.1121

[2] Swaab DF , Dubelaar EJ , Hofman MA , Scherder EJ , van Someren EJ and Verwer RW (2002) Brain aging and Alzheimer's disease; use it or lose it. Prog Brain Res 138, 343–373. 12432778 10.1016/S0079-6123(02)38086-5

[3] Binder LI , Guillozet‐Bongaarts AL , Garcia‐Sierra F and Berry RW (2005) Tau, tangles, and Alzheimer's disease. Biochim Biophys Acta 1739, 216–223. 15615640 10.1016/j.bbadis.2004.08.014

[4] Gomez‐Isla T , Hollister R , West H , Mui S , Growdon JH , Petersen RC , Parisi JE and Hyman BT (1997) Neuronal loss correlates with but exceeds neurofibrillary tangles in Alzheimer's disease. Ann Neurol 41, 17–24. 9005861 10.1002/ana.410410106

[5] Hyman BT , Phelps CH , Beach TG , Bigio EH , Cairns NJ , Carrillo MC , Dickson DW , Duyckaerts C , Frosch MP , Masliah E *et al*. (2012) National Institute on Aging‐Alzheimer's Association guidelines for the neuropathologic assessment of Alzheimer's disease. Alzheimers Dement 8, 1–13. 22265587 10.1016/j.jalz.2011.10.007 PMC3266529

[6] Santacruz K , Lewis J , Spires T , Paulson J , Kotilinek L , Ingelsson M , Guimaraes A , DeTure M , Ramsden M , McGowan E *et al*. (2005) Tau suppression in a neurodegenerative mouse model improves memory function. Science 309, 476–481. 16020737 10.1126/science.1113694 PMC1574647

[7] Andorfer C , Acker CM , Kress Y , Hof PR , Duff K and Davies P (2005) Cell‐cycle reentry and cell death in transgenic mice expressing nonmutant human tau isoforms. J Neurosci 25, 5446–5454. 15930395 10.1523/JNEUROSCI.4637-04.2005 PMC6725006

[8] Khlistunova I , Biernat J , Wang Y , Pickhardt M , von Bergen M , Gazova Z , Mandelkow E and Mandelkow EM (2006) Inducible expression of tau repeat domain in cell models of tauopathy: aggregation is toxic to cells but can be reversed by inhibitor drugs. J Biol Chem 281, 1205–1214. 16246844 10.1074/jbc.M507753200

[9] Yoshiyama Y , Higuchi M , Zhang B , Huang SM , Iwata N , Saido TC , Maeda J , Suhara T , Trojanowski JQ and Lee VM (2007) Synapse loss and microglial activation precede tangles in a P301S tauopathy mouse model. Neuron 53, 337–351. 17270732 10.1016/j.neuron.2007.01.010

[10] Shi Y , Zhang W , Yang Y , Murzin AG , Falcon B , Kotecha A , van Beers M , Tarutani A , Kametani F , Garringer HJ *et al*. (2021) Structure‐based classification of tauopathies. Nature 598, 359–363. 34588692 10.1038/s41586-021-03911-7 PMC7611841

[11] Hipp MS , Kasturi P and Hartl FU (2019) The proteostasis network and its decline in ageing. Nat Rev Mol Cell Biol 20, 421–435. 30733602 10.1038/s41580-019-0101-y

[12] Tan JM , Wong ES , Kirkpatrick DS , Pletnikova O , Ko HS , Tay SP , Ho MW , Troncoso J , Gygi SP , Lee MK *et al*. (2008) Lysine 63‐linked ubiquitination promotes the formation and autophagic clearance of protein inclusions associated with neurodegenerative diseases. Hum Mol Genet 17, 431–439. 17981811 10.1093/hmg/ddm320

[13] Cripps D , Thomas SN , Jeng Y , Yang F , Davies P and Yang AJ (2006) Alzheimer disease‐specific conformation of hyperphosphorylated paired helical filament‐tau is polyubiquitinated through Lys‐48, Lys‐11, and Lys‐6 ubiquitin conjugation. J Biol Chem 281, 10825–10838. 16443603 10.1074/jbc.M512786200

[14] Keren‐Shaul H , Spinrad A , Weiner A , Matcovitch‐Natan O , Dvir‐Szternfeld R , Ulland TK , David E , Baruch K , Lara‐Astaiso D , Toth B *et al*. (2017) A unique microglia type associated with restricting development of Alzheimer's disease. Cell 169, 1276–1290.e17. 28602351 10.1016/j.cell.2017.05.018

[15] Brown GC and St George‐Hyslop PH (2017) Deciphering microglial diversity in Alzheimer's disease. Science 356, 1123–1124. 28619900 10.1126/science.aan7893

[16] LoPresti P , Szuchet S , Papasozomenos SC , Zinkowski RP and Binder LI (1995) Functional implications for the microtubule‐associated protein tau: localization in oligodendrocytes. Proc Natl Acad Sci U S A 92, 10369–10373. 7479786 10.1073/pnas.92.22.10369 PMC40798

[17] Goedert M , Wischik CM , Crowther RA , Walker JE and Klug A (1988) Cloning and sequencing of the cDNA encoding a core protein of the paired helical filament of Alzheimer disease: identification as the microtubule‐associated protein tau. Proc Natl Acad Sci U S A 85, 4051–4055. 3131773 10.1073/pnas.85.11.4051 PMC280359

[18] Goedert M , Spillantini MG , Jakes R , Rutherford D and Crowther RA (1989) Multiple isoforms of human microtubule‐associated protein tau: sequences and localization in neurofibrillary tangles of Alzheimer's disease. Neuron 3, 519–526. 2484340 10.1016/0896-6273(89)90210-9

[19] Goedert M and Jakes R (1990) Expression of separate isoforms of human tau protein: correlation with the tau pattern in brain and effects on tubulin polymerization. EMBO J 9, 4225–4230. 2124967 10.1002/j.1460-2075.1990.tb07870.x PMC552204

[20] Dickson DW , Kouri N , Murray ME and Josephs KA (2011) Neuropathology of frontotemporal lobar degeneration‐tau (FTLD‐tau). J Mol Neurosci 45, 384–389. 21720721 10.1007/s12031-011-9589-0 PMC3208128

[21] Goedert M , Eisenberg DS and Crowther RA (2017) Propagation of tau aggregates and neurodegeneration. Annu Rev Neurosci 40, 189–210. 28772101 10.1146/annurev-neuro-072116-031153

[22] Kosik KS , Orecchio LD , Bakalis S and Neve RL (1989) Developmentally regulated expression of specific tau sequences. Neuron 2, 1389–1397. 2560640 10.1016/0896-6273(89)90077-9

[23] Janke C , Beck M , Stahl T , Holzer M , Brauer K , Bigl V and Arendt T (1999) Phylogenetic diversity of the expression of the microtubule‐associated protein tau: implications for neurodegenerative disorders. Brain Res Mol Brain Res 68, 119–128. 10320789 10.1016/s0169-328x(99)00079-0

[24] Takuma H , Arawaka S and Mori H (2003) Isoforms changes of tau protein during development in various species. Brain Res Dev Brain Res 142, 121–127. 12711363 10.1016/s0165-3806(03)00056-7

[25] Tuerde D , Kimura T , Miyasaka T , Furusawa K , Shimozawa A , Hasegawa M , Ando K and Hisanaga SI (2018) Isoform‐independent and ‐dependent phosphorylation of microtubule‐associated protein tau in mouse brain during postnatal development. J Biol Chem 293, 1781–1793. 29196605 10.1074/jbc.M117.798918 PMC5798307

[26] Ke YD , Suchowerska AK , van der Hoven J , De Silva DM , Wu CW , van Eersel J , Ittner A and Ittner LM (2012) Lessons from tau‐deficient mice. Int J Alzheimers Dis 2012, 873270. 22720190 10.1155/2012/873270 PMC3375147

[27] Harada A , Oguchi K , Okabe S , Kuno J , Terada S , Ohshima T , Sato‐Yoshitake R , Takei Y , Noda T and Hirokawa N (1994) Altered microtubule organization in small‐calibre axons of mice lacking tau protein. Nature 369, 488–491. 8202139 10.1038/369488a0

[28] Dawson HN , Ferreira A , Eyster MV , Ghoshal N , Binder LI and Vitek MP (2001) Inhibition of neuronal maturation in primary hippocampal neurons from tau deficient mice. J Cell Sci 114, 1179–1187. 11228161 10.1242/jcs.114.6.1179

[29] Fujio K , Sato M , Uemura T , Sato T , Sato‐Harada R and Harada A (2007) 14‐3‐3 proteins and protein phosphatases are not reduced in tau‐deficient mice. Neuroreport 18, 1049–1052. 17558294 10.1097/WNR.0b013e32818b2a0b

[30] Ikegami S , Harada A and Hirokawa N (2000) Muscle weakness, hyperactivity, and impairment in fear conditioning in tau‐deficient mice. Neurosci Lett 279, 129–132. 10688046 10.1016/s0304-3940(99)00964-7

[31] Lei P , Ayton S , Finkelstein DI , Spoerri L , Ciccotosto GD , Wright DK , Wong BX , Adlard PA , Cherny RA , Lam LQ *et al*. (2012) Tau deficiency induces parkinsonism with dementia by impairing APP‐mediated iron export. Nat Med 18, 291–295. 22286308 10.1038/nm.2613

[32] Cantero JL , Hita‐Yanez E , Moreno‐Lopez B , Portillo F , Rubio A and Avila J (2010) Tau protein role in sleep‐wake cycle. J Alzheimers Dis 21, 411–421. 20555133 10.3233/JAD-2010-100285

[33] Roberson ED , Scearce‐Levie K , Palop JJ , Yan F , Cheng IH , Wu T , Gerstein H , Yu GQ and Mucke L (2007) Reducing endogenous tau ameliorates amyloid beta‐induced deficits in an Alzheimer's disease mouse model. Science 316, 750–754. 17478722 10.1126/science.1141736

[34] Ittner LM , Ke YD , Delerue F , Bi M , Gladbach A , van Eersel J , Wolfing H , Chieng BC , Christie MJ , Napier IA *et al*. (2010) Dendritic function of tau mediates amyloid‐beta toxicity in Alzheimer's disease mouse models. Cell 142, 387–397. 20655099 10.1016/j.cell.2010.06.036

[35] Hong XP , Peng CX , Wei W , Tian Q , Liu YH , Yao XQ , Zhang Y , Cao FY , Wang Q and Wang JZ (2010) Essential role of tau phosphorylation in adult hippocampal neurogenesis. Hippocampus 20, 1339–1349. 19816983 10.1002/hipo.20712

[36] Kimura T , Whitcomb DJ , Jo J , Regan P , Piers T , Heo S , Brown C , Hashikawa T , Murayama M , Seok H *et al*. (2014) Microtubule‐associated protein tau is essential for long‐term depression in the hippocampus. Philos Trans R Soc Lond B Biol Sci 369, 20130144. 24298146 10.1098/rstb.2013.0144 PMC3843876

[37] Murray ME and Dickson DW (2014) Is pathological aging a successful resistance against amyloid‐beta or preclinical Alzheimer's disease? Alzheimer's Res Ther 6, 24. 25031637 10.1186/alzrt254 PMC4055017

[38] Yamada T , McGeer PL and McGeer EG (1992) Appearance of paired nucleated, Tau‐positive glia in patients with progressive supranuclear palsy brain tissue. Neurosci Lett 135, 99–102. 1371861 10.1016/0304-3940(92)90145-w

[39] Dickson DW , Bergeron C , Chin SS , Duyckaerts C , Horoupian D , Ikeda K , Jellinger K , Lantos PL , Lippa CF , Mirra SS *et al*. (2002) Office of Rare Diseases neuropathologic criteria for corticobasal degeneration. J Neuropathol Exp Neurol 61, 935–946. 12430710 10.1093/jnen/61.11.935

[40] Litvan I , Sastry N and Sonies BC (1997) Characterizing swallowing abnormalities in progressive supranuclear palsy. Neurology 48, 1654–1662. 9191782 10.1212/wnl.48.6.1654

[41] Boeve BF , Lang AE and Litvan I (2003) Corticobasal degeneration and its relationship to progressive supranuclear palsy and frontotemporal dementia. Ann Neurol 54 (Suppl 5), S15–S19. 12833363 10.1002/ana.10570

[42] Armstrong MJ , Litvan I , Lang AE , Bak TH , Bhatia KP , Borroni B , Boxer AL , Dickson DW , Grossman M , Hallett M *et al*. (2013) Criteria for the diagnosis of corticobasal degeneration. Neurology 80, 496–503. 23359374 10.1212/WNL.0b013e31827f0fd1 PMC3590050

[43] Probst A , Tolnay M , Langui D , Goedert M and Spillantini MG (1996) Pick's disease: hyperphosphorylated tau protein segregates to the somatoaxonal compartment. Acta Neuropathol 92, 588–596. 8960316 10.1007/s004010050565

[44] de Silva R , Lashley T , Strand C , Shiarli AM , Shi J , Tian J , Bailey KL , Davies P , Bigio EH , Arima K *et al*. (2006) An immunohistochemical study of cases of sporadic and inherited frontotemporal lobar degeneration using 3R‐ and 4R‐specific tau monoclonal antibodies. Acta Neuropathol 111, 329–340. 16552612 10.1007/s00401-006-0048-x

[45] Cairns NJ , Bigio EH , Mackenzie IR , Neumann M , Lee VM , Hatanpaa KJ , White CL 3rd , Schneider JA , Grinberg LT , Halliday G *et al*. (2007) Neuropathologic diagnostic and nosologic criteria for frontotemporal lobar degeneration: consensus of the Consortium for Frontotemporal Lobar Degeneration. Acta Neuropathol 114, 5–22. 17579875 10.1007/s00401-007-0237-2 PMC2827877

[46] Togo T , Sahara N , Yen SH , Cookson N , Ishizawa T , Hutton M , de Silva R , Lees A and Dickson DW (2002) Argyrophilic grain disease is a sporadic 4‐repeat tauopathy. J Neuropathol Exp Neurol 61, 547–556. 12071638 10.1093/jnen/61.6.547

[47] McKee AC , Stern RA , Nowinski CJ , Stein TD , Alvarez VE , Daneshvar DH , Lee HS , Wojtowicz SM , Hall G , Baugh CM *et al*. (2013) The spectrum of disease in chronic traumatic encephalopathy. Brain 136, 43–64. 23208308 10.1093/brain/aws307 PMC3624697

[48] Falcon B , Zivanov J , Zhang W , Murzin AG , Garringer HJ , Vidal R , Crowther RA , Newell KL , Ghetti B , Goedert M *et al*. (2019) Novel tau filament fold in chronic traumatic encephalopathy encloses hydrophobic molecules. Nature 568, 420–423. 30894745 10.1038/s41586-019-1026-5 PMC6472968

[49] Irwin DJ (2016) Tauopathies as clinicopathological entities. Parkinsonism Relat Disord 22 (Suppl 1), S29–S33. 26382841 10.1016/j.parkreldis.2015.09.020 PMC4662611

[50] Zhang Y , Wu KM , Yang L , Dong Q and Yu JT (2022) Tauopathies: new perspectives and challenges. Mol Neurodegener 17, 28. 35392986 10.1186/s13024-022-00533-z PMC8991707

[51] Olsson B , Lautner R , Andreasson U , Ohrfelt A , Portelius E , Bjerke M , Holtta M , Rosen C , Olsson C , Strobel G *et al*. (2016) CSF and blood biomarkers for the diagnosis of Alzheimer's disease: a systematic review and meta‐analysis. Lancet Neurol 15, 673–684. 27068280 10.1016/S1474-4422(16)00070-3

[52] Schoonenboom NS , Reesink FE , Verwey NA , Kester MI , Teunissen CE , van de Ven PM , Pijnenburg YA , Blankenstein MA , Rozemuller AJ , Scheltens P *et al*. (2012) Cerebrospinal fluid markers for differential dementia diagnosis in a large memory clinic cohort. Neurology 78, 47–54. 22170879 10.1212/WNL.0b013e31823ed0f0

[53] Sato C , Barthelemy NR , Mawuenyega KG , Patterson BW , Gordon BA , Jockel‐Balsarotti J , Sullivan M , Crisp MJ , Kasten T , Kirmess KM *et al*. (2018) Tau kinetics in neurons and the human central nervous system. Neuron 98, 861–864. 29772204 10.1016/j.neuron.2018.04.035 PMC6192252

[54] Janelidze S , Stomrud E , Smith R , Palmqvist S , Mattsson N , Airey DC , Proctor NK , Chai X , Shcherbinin S , Sims JR *et al*. (2020) Cerebrospinal fluid p‐tau217 performs better than p‐tau181 as a biomarker of Alzheimer's disease. Nat Commun 11, 1683. 32246036 10.1038/s41467-020-15436-0 PMC7125218

[55] Simren J , Leuzy A , Karikari TK , Hye A , Benedet AL , Lantero‐Rodriguez J , Mattsson‐Carlgren N , Scholl M , Mecocci P , Vellas B *et al*. (2021) The diagnostic and prognostic capabilities of plasma biomarkers in Alzheimer's disease. Alzheimers Dement 17, 1145–1156. 33491853 10.1002/alz.12283 PMC8359457

[56] Thijssen EH , La Joie R , Wolf A , Strom A , Wang P , Iaccarino L , Bourakova V , Cobigo Y , Heuer H , Spina S *et al*. (2020) Diagnostic value of plasma phosphorylated tau181 in Alzheimer's disease and frontotemporal lobar degeneration. Nat Med 26, 387–397. 32123386 10.1038/s41591-020-0762-2 PMC7101073

[57] Thijssen EH , La Joie R , Strom A , Fonseca C , Iaccarino L , Wolf A , Spina S , Allen IE , Cobigo Y , Heuer H *et al*. (2021) Plasma phosphorylated tau 217 and phosphorylated tau 181 as biomarkers in Alzheimer's disease and frontotemporal lobar degeneration: a retrospective diagnostic performance study. Lancet Neurol 20, 739–752. 34418401 10.1016/S1474-4422(21)00214-3 PMC8711249

[58] Maruyama M , Shimada H , Suhara T , Shinotoh H , Ji B , Maeda J , Zhang MR , Trojanowski JQ , Lee VM , Ono M *et al*. (2013) Imaging of tau pathology in a tauopathy mouse model and in Alzheimer patients compared to normal controls. Neuron 79, 1094–1108. 24050400 10.1016/j.neuron.2013.07.037 PMC3809845

[59] Xia CF , Arteaga J , Chen G , Gangadharmath U , Gomez LF , Kasi D , Lam C , Liang Q , Liu C , Mocharla VP *et al*. (2013) [(18)F]T807, a novel tau positron emission tomography imaging agent for Alzheimer's disease. Alzheimers Dement 9, 666–676. 23411393 10.1016/j.jalz.2012.11.008

[60] Hostetler ED , Walji AM , Zeng Z , Miller P , Bennacef I , Salinas C , Connolly B , Gantert L , Haley H , Holahan M *et al*. (2016) Preclinical characterization of 18F‐MK‐6240, a promising PET tracer for In vivo quantification of human neurofibrillary tangles. J Nucl Med 57, 1599–1606. 27230925 10.2967/jnumed.115.171678

[61] Walji AM , Hostetler ED , Selnick H , Zeng Z , Miller P , Bennacef I , Salinas C , Connolly B , Gantert L , Holahan M *et al*. (2016) Discovery of 6‐(Fluoro‐(18)F)‐3‐(1H‐pyrrolo[2,3‐c]pyridin‐1‐yl)isoquinolin‐5‐amine ([(18)F]‐MK‐6240): a positron emission tomography (PET) imaging agent for quantification of neurofibrillary tangles (NFTs). J Med Chem 59, 4778–4789. 27088900 10.1021/acs.jmedchem.6b00166

[62] Hall B , Mak E , Cervenka S , Aigbirhio FI , Rowe JB and O'Brien JT (2017) In vivo tau PET imaging in dementia: pathophysiology, radiotracer quantification, and a systematic review of clinical findings. Ageing Res Rev 36, 50–63. 28315409 10.1016/j.arr.2017.03.002

[63] Mueller A , Kroth H , Schieferstein H , Berndt M , Oden F , Capotosti F , Molette J , Juergens T , Darmency V , Schmitt‐Willich H *et al*. (2017) Preclinical characterization of PI‐2620, a novel tau PET tracer for detection of tau in AD and other tauopathies. Alzheimers Dement 13, 141–142.

[64] Saint‐Aubert L , Lemoine L , Chiotis K , Leuzy A , Rodriguez‐Vieitez E and Nordberg A (2017) Tau PET imaging: present and future directions. Mol Neurodegener 12, 19. 28219440 10.1186/s13024-017-0162-3 PMC5319037

[65] Tagai K , Ono M , Kubota M , Kitamura S , Takahata K , Seki C , Takado Y , Shinotoh H , Sano Y , Yamamoto Y *et al*. (2021) High‐contrast In vivo imaging of tau pathologies in Alzheimer's and non‐Alzheimer's disease tauopathies. Neuron 109, 42–58.e8. 33125873 10.1016/j.neuron.2020.09.042

[66] Braak H and Braak E (1997) Frequency of stages of Alzheimer‐related lesions in different age categories. Neurobiol Aging 18, 351–357. 9330961 10.1016/s0197-4580(97)00056-0

[67] Lowe VJ , Curran G , Fang P , Liesinger AM , Josephs KA , Parisi JE , Kantarci K , Boeve BF , Pandey MK , Bruinsma T *et al*. (2016) An autoradiographic evaluation of AV‐1451 Tau PET in dementia. Acta Neuropathol Commun 4, 58. 27296779 10.1186/s40478-016-0315-6 PMC4906968

[68] Ng KP , Pascoal TA , Mathotaarachchi S , Therriault J , Kang MS , Shin M , Guiot MC , Guo Q , Harada R , Comley RA *et al*. (2017) Monoamine oxidase B inhibitor, selegiline, reduces (18)F‐THK5351 uptake in the human brain. Alzheimer's Res Ther 9, 25. 28359327 10.1186/s13195-017-0253-y PMC5374697

[69] Gobbi LC , Knust H , Korner M , Honer M , Czech C , Belli S , Muri D , Edelmann MR , Hartung T , Erbsmehl I *et al*. (2017) Identification of three novel radiotracers for imaging aggregated tau in Alzheimer's disease with positron emission tomography. J Med Chem 60, 7350–7370. 28654263 10.1021/acs.jmedchem.7b00632

[70] Kroth H , Oden F , Molette J , Schieferstein H , Capotosti F , Mueller A , Berndt M , Schmitt‐Willich H , Darmency V , Gabellieri E *et al*. (2019) Discovery and preclinical characterization of [(18)F]PI‐2620, a next‐generation tau PET tracer for the assessment of tau pathology in Alzheimer's disease and other tauopathies. Eur J Nucl Med Mol Imaging 46, 2178–2189. 31264169 10.1007/s00259-019-04397-2 PMC6667408

[71] Ono M , Sahara N , Kumata K , Ji B , Ni R , Koga S , Dickson DW , Trojanowski JQ , Lee VM , Yoshida M *et al*. (2017) Distinct binding of PET ligands PBB3 and AV‐1451 to tau fibril strains in neurodegenerative tauopathies. Brain 140, 764–780. 28087578 10.1093/brain/aww339 PMC5837223

[72] Horie K , Salvado G , Barthelemy NR , Janelidze S , Li Y , He Y , Saef B , Chen CD , Jiang H , Strandberg O *et al*. (2023) CSF MTBR‐tau243 is a specific biomarker of tau tangle pathology in Alzheimer's disease. Nat Med 29, 1954–1963. 37443334 10.1038/s41591-023-02443-z PMC10427417

[73] Murugan NA , Nordberg A and Agren H (2018) Different positron emission tomography tau tracers bind to multiple binding sites on the tau fibril: insight from computational modeling. ACS Chem Nerosci 9, 1757–1767. 10.1021/acschemneuro.8b00093 29630333

[74] Goedert M , Yamaguchi Y , Mishra SK , Higuchi M and Sahara N (2018) Tau filaments and the development of positron emission tomography tracers. Front Neurol 9, 70. 29497399 10.3389/fneur.2018.00070 PMC5818396

[75] Falcon B , Zhang W , Murzin AG , Murshudov G , Garringer HJ , Vidal R , Crowther RA , Ghetti B , Scheres SHW and Goedert M (2018) Structures of filaments from Pick's disease reveal a novel tau protein fold. Nature 561, 137–140. 30158706 10.1038/s41586-018-0454-y PMC6204212

[76] Shi Y , Murzin AG , Falcon B , Epstein A , Machin J , Tempest P , Newell KL , Vidal R , Garringer HJ , Sahara N *et al*. (2021) Cryo‐EM structures of tau filaments from Alzheimer's disease with PET ligand APN‐1607. Acta Neuropathol 141, 697–708. 33723967 10.1007/s00401-021-02294-3 PMC8043864

[77] Fu H , Possenti A , Freer R , Nakano Y , Hernandez Villegas NC , Tang M , Cauhy PVM , Lassus BA , Chen S , Fowler SL *et al*. (2019) A tau homeostasis signature is linked with the cellular and regional vulnerability of excitatory neurons to tau pathology. Nat Neurosci 22, 47–56. 30559469 10.1038/s41593-018-0298-7 PMC6330709

[78] Berger Z , Roder H , Hanna A , Carlson A , Rangachari V , Yue M , Wszolek Z , Ashe K , Knight J , Dickson D *et al*. (2007) Accumulation of pathological tau species and memory loss in a conditional model of tauopathy. J Neurosci 27, 3650–3662. 17409229 10.1523/JNEUROSCI.0587-07.2007 PMC6672413

[79] Maeda S , Sahara N , Saito Y , Murayama M , Yoshiike Y , Kim H , Miyasaka T , Murayama S , Ikai A and Takashima A (2007) Granular tau oligomers as intermediates of tau filaments. Biochemistry 46, 3856–3861. 17338548 10.1021/bi061359o

[80] Lasagna‐Reeves CA , Castillo‐Carranza DL , Sengupta U , Clos AL , Jackson GR and Kayed R (2011) Tau oligomers impair memory and induce synaptic and mitochondrial dysfunction in wild‐type mice. Mol Neurodegener 6, 39. 21645391 10.1186/1750-1326-6-39 PMC3224595

[81] Ono M , Komatsu M , Ji B , Takado Y , Shimojo M , Minamihisamatsu T , Warabi E , Yanagawa T , Matsumoto G , Aoki I *et al*. (2022) Central role for p62/SQSTM1 in the elimination of toxic tau species in a mouse model of tauopathy. Aging Cell 21, e13615. 35662390 10.1111/acel.13615 PMC9282839

[82] Patterson KR , Remmers C , Fu Y , Brooker S , Kanaan NM , Vana L , Ward S , Reyes JF , Philibert K , Glucksman MJ *et al*. (2011) Characterization of prefibrillar Tau oligomers in vitro and in Alzheimer disease. J Biol Chem 286, 23063–23076. 21550980 10.1074/jbc.M111.237974 PMC3123074

[83] Schmidt MF , Gan ZY , Komander D and Dewson G (2021) Ubiquitin signalling in neurodegeneration: mechanisms and therapeutic opportunities. Cell Death Differ 28, 570–590. 33414510 10.1038/s41418-020-00706-7 PMC7862249

[84] Chu TT , Gao N , Li QQ , Chen PG , Yang XF , Chen YX , Zhao YF and Li YM (2016) Specific knockdown of endogenous tau protein by peptide‐directed ubiquitin‐proteasome degradation. Cell Chem Biol 23, 453–461. 27105281 10.1016/j.chembiol.2016.02.016

[85] Silva MC , Ferguson FM , Cai Q , Donovan KA , Nandi G , Patnaik D , Zhang T , Huang HT , Lucente DE , Dickerson BC *et al*. (2019) Targeted degradation of aberrant tau in frontotemporal dementia patient‐derived neuronal cell models. Elife 8, e45457. 30907729 10.7554/eLife.45457 PMC6450673

[86] Takahashi D , Moriyama J , Nakamura T , Miki E , Takahashi E , Sato A , Akaike T , Itto‐Nakama K and Arimoto H (2019) AUTACs: cargo‐specific degraders using selective autophagy. Mol Cell 76, 797–810.e10. 31606272 10.1016/j.molcel.2019.09.009

[87] Fu R , Shen Q , Xu P , Luo JJ and Tang Y (2014) Phagocytosis of microglia in the central nervous system diseases. Mol Neurobiol 49, 1422–1434. 24395130 10.1007/s12035-013-8620-6 PMC4012154

[88] Leyns CEG , Ulrich JD , Finn MB , Stewart FR , Koscal LJ , Remolina Serrano J , Robinson GO , Anderson E , Colonna M and Holtzman DM (2017) TREM2 deficiency attenuates neuroinflammation and protects against neurodegeneration in a mouse model of tauopathy. Proc Natl Acad Sci U S A 114, 11524–11529. 29073081 10.1073/pnas.1710311114 PMC5663386

[89] Maeda J , Zhang MR , Okauchi T , Ji B , Ono M , Hattori S , Kumata K , Iwata N , Saido TC , Trojanowski JQ *et al*. (2011) In vivo positron emission tomographic imaging of glial responses to amyloid‐beta and tau pathologies in mouse models of Alzheimer's disease and related disorders. J Neurosci 31, 4720–4730. 21430171 10.1523/JNEUROSCI.3076-10.2011 PMC3251921

[90] Ishikawa A , Tokunaga M , Maeda J , Minamihisamatsu T , Shimojo M , Takuwa H , Ono M , Ni R , Hirano S , Kuwabara S *et al*. (2018) In vivo visualization of tau accumulation, microglial activation, and brain atrophy in a mouse model of tauopathy rTg4510. J Alzheimers Dis 61, 1037–1052. 29332041 10.3233/JAD-170509

[91] Fairley LH , Sahara N , Aoki I , Ji B , Suhara T , Higuchi M and Barron AM (2021) Neuroprotective effect of mitochondrial translocator protein ligand in a mouse model of tauopathy. J Neuroinflammation 18, 76. 33740987 10.1186/s12974-021-02122-1 PMC7980620

[92] Wu T , Dejanovic B , Gandham VD , Gogineni A , Edmonds R , Schauer S , Srinivasan K , Huntley MA , Wang Y , Wang TM *et al*. (2019) Complement C3 is activated in human AD brain and is required for neurodegeneration in mouse models of amyloidosis and tauopathy. Cell Rep 28, 2111–2123.e6. 31433986 10.1016/j.celrep.2019.07.060

[93] Hansen DV , Hanson JE and Sheng M (2018) Microglia in Alzheimer's disease. J Cell Biol 217, 459–472. 29196460 10.1083/jcb.201709069 PMC5800817

[94] Brown GC and Neher JJ (2014) Microglial phagocytosis of live neurons. Nat Rev Neurosci 15, 209–216. 24646669 10.1038/nrn3710

[95] Lemke G (2019) How macrophages deal with death. Nat Rev Immunol 19, 539–549. 31019284 10.1038/s41577-019-0167-y PMC6733267

[96] Zagorska A , Traves PG , Lew ED , Dransfield I and Lemke G (2014) Diversification of TAM receptor tyrosine kinase function. Nat Immunol 15, 920–928. 25194421 10.1038/ni.2986 PMC4169336

[97] Scott RS , McMahon EJ , Pop SM , Reap EA , Caricchio R , Cohen PL , Earp HS and Matsushima GK (2001) Phagocytosis and clearance of apoptotic cells is mediated by MER. Nature 411, 207–211. 11346799 10.1038/35075603

[98] Lemke G (2013) Biology of the TAM receptors. Cold Spring Harb Perspect Biol 5, a009076. 24186067 10.1101/cshperspect.a009076 PMC3809585

[99] Huang Y , Happonen KE , Burrola PG , O'Connor C , Hah N , Huang L , Nimmerjahn A and Lemke G (2021) Microglia use TAM receptors to detect and engulf amyloid beta plaques. Nat Immunol 22, 586–594. 33859405 10.1038/s41590-021-00913-5 PMC8102389

[100] Sobue A , Komine O , Hara Y , Endo F , Mizoguchi H , Watanabe S , Murayama S , Saito T , Saido TC , Sahara N *et al*. (2021) Microglial gene signature reveals loss of homeostatic microglia associated with neurodegeneration of Alzheimer's disease. Acta Neuropathol Commun 9, 1. 33402227 10.1186/s40478-020-01099-x PMC7786928

[101] Maeda J , Minamihisamatsu T , Shimojo M , Zhou X , Ono M , Matsuba Y , Ji B , Ishii H , Ogawa M , Akatsu H *et al*. (2021) Distinct microglial response against Alzheimer's amyloid and tau pathologies characterized by P2Y12 receptor. Brain Commun 3, fcab011. 33644757 10.1093/braincomms/fcab011 PMC7901060

[102] Cserep C , Posfai B , Lenart N , Fekete R , Laszlo ZI , Lele Z , Orsolits B , Molnar G , Heindl S , Schwarcz AD *et al*. (2020) Microglia monitor and protect neuronal function through specialized somatic purinergic junctions. Science 367, 528–537. 31831638 10.1126/science.aax6752

[103] Cummings J , Lee G , Ritter A and Zhong K (2018) Alzheimer's disease drug development pipeline: 2018. Alzheimers Dement 4, 195–214. 10.1016/j.trci.2018.03.009 PMC6021548 29955663

[104] Kimura T , Ono M , Seki C , Sampei K , Shimojo M , Kawamura K , Zhang MR , Sahara N , Takado Y and Higuchi M (2022) A quantitative in vivo imaging platform for tracking pathological tau depositions and resultant neuronal death in a mouse model. Eur J Nucl Med Mol Imaging 49, 4298–4311. 35798978 10.1007/s00259-022-05898-3

[105] Shimojo M , Higuchi M , Suhara T and Sahara N (2015) Imaging multimodalities for dissecting Alzheimer's disease: advanced technologies of positron emission tomography and fluorescence imaging. Front Neurosci 9, 482. 26733795 10.3389/fnins.2015.00482 PMC4686595

[106] Sato H , Takado Y , Toyoda S , Tsukamoto‐Yasui M , Minatohara K , Takuwa H , Urushihata T , Takahashi M , Shimojo M , Ono M *et al*. (2021) Neurodegenerative processes accelerated by protein malnutrition and decelerated by essential amino acids in a tauopathy mouse model. Sci Adv 7, eabd5046. 34678069 10.1126/sciadv.abd5046 PMC8535828

[107] Baker SL , Provost K , Thomas W , Whitman AJ , Janabi M , Schmidt ME , Timmers M , Kolb HC , Rabinovici GD and Jagust WJ (2021) Evaluation of [(18)F]‐JNJ‐64326067‐AAA tau PET tracer in humans. J Cereb Blood Flow Metab 41, 3302–3313. 34259071 10.1177/0271678X211031035 PMC8669274

[108] Sahara N and Yanai R (2023) Limitations of human tau‐expressing mouse models and novel approaches of mouse modeling for tauopathy. Front Neurosci 17, 1149761. 37152607 10.3389/fnins.2023.1149761 PMC10157230

[109] Goodwin LO , Splinter E , Davis TL , Urban R , He H , Braun RE , Chesler EJ , Kumar V , van Min M , Ndukum J *et al*. (2019) Large‐scale discovery of mouse transgenic integration sites reveals frequent structural variation and insertional mutagenesis. Genome Res 29, 494–505. 30659012 10.1101/gr.233866.117 PMC6396414

[110] Gamache JE , Kemper L , Steuer E , Leinonen‐Wright K , Choquette JM , Hlynialuk C , Benzow K , Vossel KA , Xia W , Koob MD *et al*. (2020) Developmental pathogenicity of 4‐repeat human tau is lost with the P301L mutation in genetically matched tau‐transgenic mice. J Neurosci 40, 220–236. 31685653 10.1523/JNEUROSCI.1256-19.2019 PMC6939485

[111] Chaney A , Williams SR and Boutin H (2019) In vivo molecular imaging of neuroinflammation in Alzheimer's disease. J Neurochem 149, 438–451. 30339715 10.1111/jnc.14615 PMC6563454

[112] Ji B , Ono M , Yamasaki T , Fujinaga M , Zhang MR , Seki C , Aoki I , Kito S , Sawada M , Suhara T *et al*. (2021) Detection of Alzheimer's disease‐related neuroinflammation by a PET ligand selective for glial versus vascular translocator protein. J Cereb Blood Flow Metab 41, 2076–2089. 33557690 10.1177/0271678X21992457 PMC8327108

[113] Janssen B , Vugts DJ , Funke U , Molenaar GT , Kruijer PS , van Berckel BN , Lammertsma AA and Windhorst AD (2016) Imaging of neuroinflammation in Alzheimer's disease, multiple sclerosis and stroke: recent developments in positron emission tomography. Biochim Biophys Acta 1862, 425–441. 26643549 10.1016/j.bbadis.2015.11.011

[114] Sahara N , Maeda J , Ishikawa A , Tokunaga M , Suhara T and Higuchi M (2018) Microglial activation during pathogenesis of tauopathy in rTg4510 mice: implications for the early diagnosis of tauopathy. J Alzheimers Dis 64, S353–S359. 29865054 10.3233/JAD-179933

[115] Mishra SK , Yamaguchi Y , Higuchi M and Sahara N (2020) Pick's Tau fibril shows multiple distinct PET probe binding sites: insights from computational modelling. Int J Mol Sci 22, 349. 33396273 10.3390/ijms22010349 PMC7796283

